# Prevalence and factors associated with urogenital schistosomiasis among primary school children in barrage, Magba sub-division of Cameroon

**DOI:** 10.1186/s12889-017-4539-6

**Published:** 2017-07-03

**Authors:** Anna Longdoh Njunda, Emmanuel Ngala Ndzi, Jules Clement Nguedia Assob, Henri-Lucien Fawmno Kamga, Emmanuel Tebit Kwenti

**Affiliations:** 1 Department of Medical Laboratory Sciences, University of Buea, P.B, 63 Buea, Cameroon; 2 Department of Microbiology and Parasitology, University of Buea, P.B, 63 Buea, Cameroon; 3 Department of Public Health and Hygiene, University of Buea, P.B, 63 Buea, Cameroon; 4 Department of Medical Laboratory Sciences, University of Bamenda, P.B, 39 Bamenda, Cameroon

**Keywords:** Urogenital schistosomiasis, Prevalence, Children, Associated factors, Barrage, Cameroon

## Abstract

**Background:**

The purpose of this study was to determine the prevalence and intensity as well as the factors associated with urogenital schistosomiasis (US) in Barrage, a rural community around the Mape΄ dam, in the West region of Cameroon not previously documented for transmission.

**Methods:**

In this cross sectional parasitological survey, 382 children were enrolled from three primary schools in the study area between March and May 2016. A semi-structured questionnaire was used to collect information on demographics, clinical and predisposing factors. The syringe filtration technique was used to analyse urine samples. Samples with visible or gross haematuria were recorded prior to filtration. The Pearson chi-square, the student T-test and logistic regression were all performed as part of the statistical analyses.

**Results:**

The overall prevalence of US was 41.1% (95% CI: 36.1–46.2). Infection was more common in children below 10 years (*p* = 0.009), in males (*p* = 0.029), and in children who frequently come into contact with water from the dam (*p* < 0.001). Furthermore, US was more common in children attending Ecole Public (EP) Manbonko Bord (81.1%, *p* < 0.001) which is very close to the dam and in children from a fishing background (80.9%, *p* < 0.001). On the contrary, knowledge about schistosomiasis was not observed to be associated with prevalence. In this study, the intensity of infection was observed to be higher in children below 10 years (*p* < 0.001), in males (*p* = 0.001), and in children attending EP Manbonko Bord (*p* < 0.001). The intensity of infection was also highest in children presenting with haematuria (*p* < 0.001). Frequent contact with water from the dam and having parents whose occupation was fishing were identified as the associated factors for US.

**Conclusion:**

A high prevalence of US was observed in school-aged children in the study area especially in those attending EP Manbonko Bord. Limiting contact with water from the dam, control of the snail intermediate host, provision of portable water and mass treatment of the entire population are proposed as some of the measures to reduce and eventually eliminate transmission in the area.

## Background

Schistosomiasis or bilharzia is a parasitic disease caused by blood-dwelling flukes of the genius *Schistosoma*. Schistosomiasis affects 240 million people worldwide [1]. Every year, an estimated 200,000 people die from the disease [2, 3]. The infection is more prevalent in poor communities without portable water and adequate sanitation, characteristic of most developing countries in Africa, Asia and South America. A great majority (85%) of cases occur in Africa [4]. An estimated 700 million people, in more than 70 tropical and sub-tropical countries, live in areas where the disease is common [2]. In tropical countries, schistosomiasis is second only to malaria as the most important parasitic disease with the greatest economic impact [5].

Schistosomiasis can be grouped into two categories based on the organ affected – urogenital schistosomiasis and intestinal schistosomiasis. Urogenital schistosomiasis is caused by *Schistosoma haematobium* and intestinal schistosomiasis by any of the following organisms namely; *S. guineensis*, *S. intercalatum*, *S. mansoni*, *S. japonicum*, and *S. mekongi*. Among the schistosomes, *S. haematobium* is the deadliest [6]. It infects over 112 million people annually in sub-Saharan Africa Alone [6].

Schistosomiasis is highly endemic in Cameroon but its distribution is uneven. Prevalence is higher in the northern regions. Unlike the northern regions where infection is widespread, transmission in the southern regions is focalized to areas where the snail intermediate host are found [7]. Prevalence of schistosomiasis in the country ranges from 1.7 to 55.5% [8–12] and rural communities are the most affected. In the west region where this study was performed, the prevalence is estimated at 3.98% [10]. Species of schistosomes endemic in Cameroon include *S. haematobium*, *S. mansoni* and *S. intercalatum* [13, 14], with the former being the most prevalent. Schistosomiasis in Cameroon affects mainly school-aged children [15]. The development of dams for hydroelectric power, irrigation canals and lack of portable drinking water are some of the factors that have greatly contributed to the high prevalence of schistosomiasis in the country [16]. The variation in the distribution of schistosomiasis in Cameroon also extends to the snail intermediate host; *Bulinus globusus* and *B. senegalensis* are the most common hosts transmitting *S. haematobium* in the northern half of the country meanwhile *B. truncatus* is the principal host transmitting *S. haematobium* in the southern half [17].

Control of schistosomiasis in Cameroon is embedded in the National Control Program for NTDs and generally involves the distribution of several drugs to school-aged children including membendazole, albendazole, ivermectin and praziquantel, with the latter distributed only in high endemic areas of schistosomiasis [10, 18]. Despite considerable efforts to scale-up activities to encompass all the regions in country, coverage still remains poor in rural areas, partly as a result of the unavailability of documented transmission [18]. For example, in Barrage, a rural community in Magba subdivision in the West region of Cameroon, all the factors that favour transmission of schistosomiasis are present including the presence of a dam to generate electricity, irrigation scheme, absence of portable water and the snail intermediate host, but no documented transmission of schistosomiasis in the area exists. This study was aimed at determining the prevalence and intensity of urogenital schistosomiasis as well as determine the associated factors for infection in the study area, so as to generate baseline data that may raise awareness of the impending problem and also mobilize control efforts directed towards the study area.

## Methods

### Study design and duration

This was a cross sectional parasitological survey involving school-aged (7–14 years) children recruited between March and May 2016 in Barrage.

### Study area

**Fig. 1:**
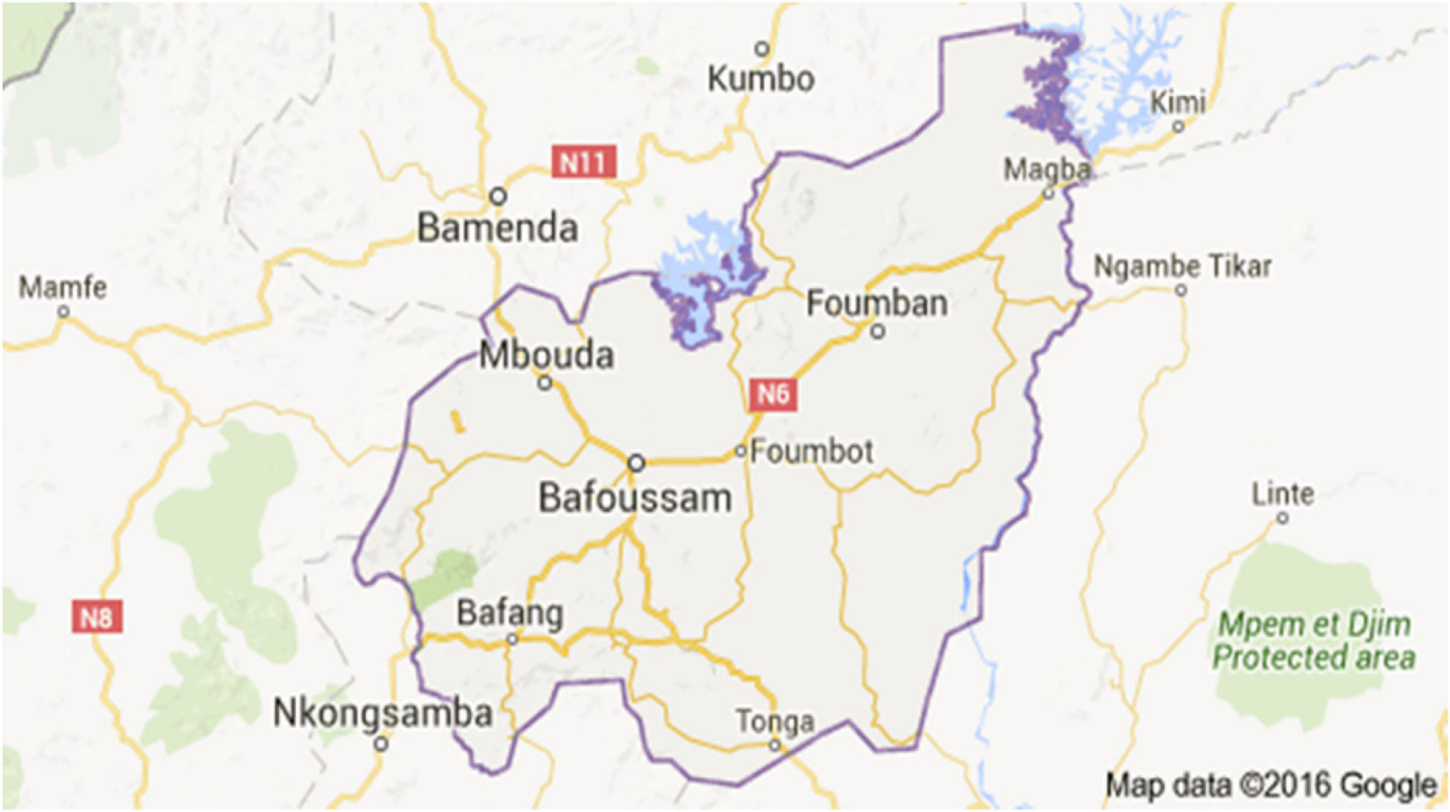
Map of the West region of Cameroon

Barrage is a village in Magba subdivision (5°57′N 11°13′E) in the West region of Cameroon. Barrage is about 14 km from Magba town and lies north east to it along the road to Banyo (Fig. 1). Barrage is inhabited by five main ethnic groups; the Bamoums, Junkums, Musko, Kotokos, and Arabs. Farming and fishing are the chief activities and source of livelihood for the inhabitants of the area. Barrage has a dam (Mapeʹ Dam) that generates electricity for the entire Magba subdivision and its environs. This dam serves as the main source of water for fishing and domestic use for most of the inhabitants of the area; there is also a stream situated further away (about 20 km) from the dam, that serves the community of Matta village. The waters from the dam and the accessory river are also used for irrigation purposes in the cultivation of tomatoes, vegetables and other cash crops characteristic of the area. The area is prone to high average temperatures of about 24–27 °C which favour high release of cercariae into the waters. Barrage has 4 primary schools namely Ecole Public (EP) Matta Barrage, EP Matta village, EP Manbonko Bord and EP Manbonko village. EP Matta Barrage, EP Matta village, EP Manbonko Bord are the schools with the highest enrollment with 996, 331 and 151 pupils respectively.

### Study population

School-aged (7–14 years) children were enrolled from 3 of the 4 primary schools in the study area namely; EP Matta-Barrage, EP Matta village, and EP Manbonko Bord randomly selected. Participants were selected by first stratifying the children according to the different grades (1–6) and then randomly selecting equal proportion of children in the different grades. This was done for all the three schools. The final proportion of participants recruited from the different schools was proportional to the number of children attending the different schools. Once selected, the children were provided with an informed consent form to be filled by their parents back at home and to be returned the following day.

### Sample size estimation

Using the following formula for sample size calculation [19];

$$ \mathrm{n}=\frac{Z^2 x\  p\left(1- p\right)}{e^2} $$

Z = 1.96.

p = prevalence of urogenital schistosomiasis = 40.27% [9].

e = error rate = 0.05

$$ \mathrm{n}=\frac{1.96^2 x\ 0.403\left(1-0.403\right)}{0.05^2}=370 $$

It was estimated that a sample of at least 370 school-aged children will be required for this study.

Overall, 413 children were approached to accommodate for loss of sample due to the fact that not all the children will return the signed informed consent form and also the inability of all the children to provide urine sample at the time of specimen collection.

### Sample collection and processing

About 20 ml of terminally voided urine sample was collected from consented participants into sterile dry leak-proof transparent urine cups. Collection was done during their break (i.e. between 10.00 and 14.00/h) when the children had exercised to ensure maximum excretion of eggs. The Urine samples were then transported to the laboratory of the Ngounso Baptist Health Center where they were examined using the syringe filtration technique [20] by a team of trained laboratory technicians. Haematuria was determined by visual observation of urine samples and reagent strips.

### Socio-demographic and risk factor assessment

A semi-structured questionnaire containing questions on the known risk factors of schistosomiasis was administered verbally to the participants by members of the research team in the local language. For children who could not express themselves, their parents or guardian were contacted to fill the questionnaire, which was also done verbally by members of the research team. The questionnaire addressed socio-demographic information, risk factors of urogenital schistosomiasis such as source of water for domestic use, swimming or playing in the dam, not wearing shoes regularly, knowledge about schistosomiasis (including the cause, mode of transmission, symptoms of infection and how infection could be prevented) etc., and clinical presentation such as abdominal pain, pain on urination and the passage of bloody urine (haematuria) etc. The associated factors were measured based on the responses of the children, parents or guardians.

### Data management and statistical analysis

Questionnaires were checked for the correct use of codes and completeness. Data was coded and entered into excel spreadsheet and analysed using the Stata® version 12.1 software (StataCorp LP, Texas, USA). The statistical tests performed included the Pearson’s Chi-square for comparison of proportions, student T test and one-way ANOVA for comparison of group means, and logistic regression analysis for risk factors for schistosomiasis. Statistical significance was set at *p* < 0.05.

## Results

Out of the 413 children approached, 382 provided urine sample and were included into the study. Those excluded did not differ from those included in the study in terms of their age (*p* = 0.285) or gender (*p* = 0.604). The sex ratio (M/F) of the participants was 1 with 191 males and 191 females. The ages of the participants ranged between 7 and 14 years (mean ± SD = 10.2 ± 1.9). The majority of the participants were recruited from EP Matta Barrage.

The most frequent clinical presentation observed in the children was passage of bloody urine or haematuria 125 (32.7%) (Table 1).

**Table 1:** Clinical and demographic characteristics of the study population

Parameter			n (%)
Age		<10	143 (37.4)
		≥10	239 (62.6)
		Total	382
Gender		M	191 (50.0)
		F	191 (50.0)
		Total	382
School		EP Matta Barrage	220 (57.6)
		EP Matta village	109 (28.5)
		EP Manbonko Bord	53 (13.9)
		Total	382
Clinical presentation	Pain on urination	Yes	64 (16.8)
		No	318 (83.2)
		Total	382
	Increased frequency of urination	Yes	91 (23.8)
		No	291 (76.2)
		Total	382
	Haematuria	Yes	125 (32.7)
		No	257 (67.3)
		Total	382
Frequent contact with dam water		Yes	221 (57.9)
		No	161 (42.1)
		Total	382

A majority (57.9%) of the participants admitted to be using water from the dam for bathing and other household chores (Table 1).

Among the 382 participants, 157 were shedding eggs of *Schistosoma haematobium* in their urine, giving an overall prevalence of 41.1% (95% CI: 36.1–46.2). The prevalence of urogenital schistosomiasis (US) was significantly higher in children below 10 years (*p* = 0.009), and in males (*p* = 0.029) (Table 2). The prevalence of US was highest in children recruited from EP Manbonko Bord. A significant association was observed between prevalence of US and the primary school attended (*p* < 0.001) (Table 2). The prevalence of US was highest in children whose parents’ main occupation was fishing and lowest in children whose parents were civil servants. A significant association was observed between prevalence of US and the occupation of the parents (*p* < 0.001). The prevalence of US was significantly higher in children who had frequent contact with water from the dam (*p* < 0.001). No significant association was observed between prevalence of US and knowledge on the disease (*p* = 0.755).

**Table 2:** Univariate analysis of some factors associated urogenital schistosomiasis in the study population

Study parameter			n	Positive n (%)	Chi-square	*p*-value
Age		<10	143	71 (49.7)	6.903	0.009
		≥10	239	86 (36.0)		
Gender		M	191	89 (46.6)	4.769	0.029
		F	191	68 (35.6)		
School		EP Matta Barrage	220	108 (49.1)	97.940	<0.001
		EP Matta village	109	6 (5.5)		
		EP Manbonko Bord	53	43 (81.1)		
Clinical presentation	Pain on urination	Yes	64	44 (68.8)	24.281	<0.001
		No	318	113 (35.5)		
	Increased frequency of urination	Yes	91	48 (52.8)	6.695	0.010
		No	291	109 (37.5)		
	Haematuria	Yes	125	121 (96.8)	238.124	<0.001
		No	257	36 (14.0)		
Frequent contact with water from the dam		Yes	221	156 (70.6)	188.359	<0.001
		No	161	1 (0.6)		
Occupation of parents		Farming	230	61 (26.5)	67.468	<0.001
		Fishing	68	55 (80.9)		
		Trading	77	38 (49.4)		
		Employed^a^	7	3 (42.9)		
Knowledge about schistosomiasis		Yes	191	77 (40.3)	0.097	0.755
		No	191	80 (41.9)		

^a^this group included participants that were serving under the government and therefore had a stable source of income

A significant association was observed between prevalence of US and pain on urination (*p* < 0.001), increased frequency of urination (0.010), and haematuria (*p* < 0.001) (Table 2).

Overall, the mean parasite load was 154.7 (±8.3) ova/10 ml of urine. The intensity of infection was higher in children below 10 years (*p* < 0.001), and in males (*p* = 0.001) (Table 3). Children attending EP Manbonko Bord had the highest parasite load compared to children from the other schools (*p* < 0.001). The intensity of infection was highest in children who presented with haematuria compared to the other clinical presentations (*p* < 0.001) (Table 3).

**Table 3:** Distribution of the intensity of infection in the study population stratified according to age, gender, school attended and clinical presentation

Parameter		Number positive	Average ova count (±SD)	*p*-value
Age (years)	<10	71	156.4 (±12.3)	<0.001
	≥10	86	144.7 (±10.5)	
Gender	M	89	154.6 (±11.1)	0.001
	F	68	149.2 (±10.1)	
School	EP Matta Barrage	108	151.8 (±9.7)	<0.001
	EP Matta village	6	94.9 (±10.4)	
	EP Manbonko Bord	43	167.6 (±10.2)	
Clinical presentation	Pain on urination	44	151.3 (±11.1)	<0.001
	Increased frequency of urination	48	80.3 (±6.7)	
	Haematuria	121	177.9 (±9.8)	

Multinomial logistic regression analysis revealed that use of water from the dam and having a parent whose occupation was fishing were the factors associated with *Schistosoma haematobium* infection (Table 4).

**Table 4:** Logistic regression analysis of some factors associated with urogenital schistosomiasis in the study population

Parameter		OR (95% CI)	*p*-value
School	EP Manbongko bord	0.43 (0.11–1.84)	0.276
	EP Matta Barrage	0.54 (0.16–1.76)	0.318
	EP Matta village	1.00	
Gender	Female	1.33 ().72–2.45)	0.366
	Male	1.00	
Age	<10 years	1.18 (0.56–2.45)	0.665
	≥10 years	1.00	
Knowledge about schistosomiasis	Yes	0.98 (0.48–2.02)	0.959
	No	1.00	
Frequent contact with water from dam	Yes	248.4 (35.97–2123.32)	<0.001
	No	1.00	
Wear shoes regularly	No	0.98 (0.44–2.18)	0.967
	Yes	1.00	
Occupation of parents	Employed	0.16 (0.02–5.31)	0.307
	Farming	1.49 (0.74–3.01)	0.270
	Fishing	0.32 (0.12–0.82)	0.019
	Trading	1.00	

## Discussion

The prevalence of urogenital schistosomiasis (US) observed in this study was 41.1%. The prevalence observed was similar to that reported in other foci in the South West region: 40.27% in Munyenge [9] and 41.3% in Marumba [21]. The prevalence of US observed in this study was however higher compared to the overall prevalence of schistosomiasis reported in the West Region [10] and in other areas of Cameroon: 1.7% in Kékem [12], 32.1% in Kumba [22] and 22.9% in Maroua [15]. These areas have regularly been targeted for control of schistosomiasis and geohelminths which may have accounted to the lower prevalence of schistosomiasis reported. Another reason that might have accounted for the lower prevalence of schistosomiasis observed in these studies compared to the current study could be the differences in the geographical settings; these studies were performed in urban and semi-urban settings meanwhile the current study was performed in a rural setting. Higher prevalence of schistosomiasis in rural areas have also been reported in other parts of the country [9, 16, 23, 24]. As noted by Njiokou et al. [23], urbanization reduces transmission points and the creation of modern water points limits the frequency of human water contacts.

In this study, haematuria, increased frequency of urination and pain on urination were identified as the most common symptom of infection with *Schistosoma haematobium* which is not very different from the findings in the study performed by Saotoing et al. [15]. Compared to the other clinical presentations of US, children with haematuria had significantly higher parasite load (*p* < 0.001). This suggest that higher parasite load is needed to cause the damage that leads to extrusion of blood into the bladder as seen during urination.

In the current study, schistosomiasis was significantly more common in males. The higher prevalence in males could be attributed to the higher tendencies of water contact through swimming, playing, fishing and engagement in other activities like the making of burnt bricks along infested water bodies besides the primary domestic activities of washing and fetching water which exposes both sexes to infection. The finding of higher prevalence of schistosomiasis among males in this study is in line with other studies performed elsewhere [14, 25–27]. These findings are however contradictory to the study by Ntonifor et al. [9] and Ndamukong et al. [24] in which infestation with schistosomes were more common in females. The difference could be attributed to the fact that boys compared to girls were the ones who frequently come into contact with infested water either through playing, bathing, or fishing in the current study.

Infection with *S. haematobium* was observed to be more common in children below 10 years of age compared to children aged 10 years and above. The finding of lower prevalence of schistosomiasis in older children could be attributed to the fact that as the children grow older, they become more aware and begin to follow the basic rules of hygiene limiting their contact with infested water bodies. The finding of higher prevalence in children below 10 years is contradictory to the study by Ntonifor et al., [9] in which infestation was more common in older children. The difference between their studies and ours could be attributed to the different behavioural pattern and cultural practices of the different populations. Fishing is one of the principle activity of the inhabitants of barrage, and younger children are frequently expose as they come in contact with infested water through fishing, meanwhile in the study of Ntonifor et al. [9], the principal activity of the inhabitants was farming which explains why older children were more infected.

Urogenital schistosomiasis was observed to be more common in EP Manbonko Bord (*p* < 0.001). The high prevalence of US in this school was probably due to its close proximity to the dam where the children frequently move to the dam within a few minutes. Moreover compared to the other schools, this school was poorly constructed without cemented floors which needed to be constantly watered to avoid dust, and the dam was the only source of water closest to the school. All these increases the exposure of the children to infection. Similar observations have been reported in studies performed elsewhere [9, 28]. The prevalence of schistosomiasis was lowest in EP Matta village. This low prevalence in this school is understandably so because Matta village is about 2 km away from the dam, and the inhabitants tend to use water from the stream present in the area. This finding affirms the fact that closeness of the school to the dam has a bearing on the prevalence of schistosomiasis. The few children infected with *S. haematobium* in this school were probably those who went for fishing at the dam. The finding of significant association between prevalence of schistosomiasis and the school attended is contrary to the study performed in Kékem [12] in which no significant association was observed. This difference could be attributed to the low prevalence of US in Kékem. This therefore shows how control programs could be beneficial provided coverage was extensive especially in rural areas.

The mean parasite load was 154.7ova/10 ml of urine. The intensity of infection was significantly higher in children below 10 years. The intensity of infection was significantly higher in males. This may be a reflection of the frequency of contact with infested water; as mentioned earlier, in this study, children below 10 years and males were the groups that frequently come into contact with infested water and therefore tend to be infected on multiple occasion. This hypothesis is supported by the observation that in this study, the intensity of infection was higher in children attending EP Manbonko Bord, which was the closest school to the dam. The finding of higher parasite load in males in this study is contrary to the study by Nkengazong et al. [21] in which no significant difference was observed in the intensity of infection between males and females. The difference between this study and ours could be attributed to differences in the exposure between the two populations; unlike in our study where males were more exposed than females through fishing activity, in the study by Nkengazong et al. [21], the rate of exposure was similar between males and females since they frequent the lake in the area equally to get water for their domestic use.

In this study, frequent contact with water from the dam and having parents whose occupation was fishing were identified as the associated factors for infection with *Schistosoma haematobium*. Many inhabitants in Cameroon especially in rural areas have limited access to portable water for domestic use leaving them with the option of using natural water bodies such as lakes, rivers, ponds and dams which may be infested with the parasite. As the dam in Barrage was the main source of water for fishing and domestic use, it is more likely that the children became infected as the come in contact with the infested water. The finding of higher rate of infections in children whose parents had fishing as their main occupation is further incriminating to the dam as the source of the infections. Development of irrigation schemes and other water bodies have also been incriminated in the increase of transmission of schistosomiasis in other areas in Cameroon [9, 16, 29].

In this study, children were recruited only from the three schools mentioned above. The findings are therefore not generalizable to preschool and school-aged children not attending these primary schools. This constitute a major limitation to this study. Studies designed to target children at the level of the communities will therefore be needed to provide a clearer picture of urogenital schistosomiasis in the study area. Furthermore, this study relied only on statistical methods to establish the factors associated with schistosomiasis in the study population, studies will therefore be needed to confirm that the dam is infested with snails which harbour the infective larvae of the parasite.

## Conclusion

This study shows a high prevalence of 41.1% for urogenital schistosomiasis in the study area putting Barrage within the WHO [30] classification as endemic. Prevalence of urogenital schistosomiasis was observed to be higher in children below 10 years and in males. Furthermore prevalence was observed to be significantly higher in children attending EP Manbonko Bord which is very close to the dam and in children whose parents’ main occupation was fishing. The intensity of infection was significantly higher in children below 10 years, in males and in children attending EP Manbonko Bord. Frequent contact with water from the dam and having parents whose main occupation was fishing were all observed to be the independent predictors of infection with *Schistosoma haematobium*. To effectively control transmission of the disease in the area will entails limiting contact with water from the dam, eliminating the snail intermediate host, educating the population and mass drug administration of praziquantel to the entire population of Barrage.
